# A Chemical Analog of Curcumin as an Improved Inhibitor of Amyloid Abeta Oligomerization

**DOI:** 10.1371/journal.pone.0031869

**Published:** 2012-03-19

**Authors:** Robert A. Orlando, Amanda M. Gonzales, Robert E. Royer, Lorraine M. Deck, David L. Vander Jagt

**Affiliations:** 1 Department of Biochemistry and Molecular Biology, University of New Mexico, School of Medicine, Albuquerque, New Mexico, United States of America; 2 Department of Chemistry and Chemical Biology, University of New Mexico, Albuquerque, New Mexico, United States of America; Brigham and Women's Hospital, Harvard Medical School, United States of America

## Abstract

Amyloid-like plaques are characteristic lesions defining the neuropathology of Alzheimer's disease (AD). The size and density of these plaques are closely associated with cognitive decline. To combat this disease, the few therapies that are available rely on drugs that increase neurotransmission; however, this approach has had limited success as it has simply slowed an imminent decline and failed to target the root cause of AD. Amyloid-like deposits result from aggregation of the Aβ peptide, and thus, reducing amyloid burden by preventing Aβ aggregation represents an attractive approach to improve the therapeutic arsenal for AD. Recent studies have shown that the natural product curcumin is capable of crossing the blood-brain barrier in the CNS in sufficient quantities so as to reduce amyloid plaque burden. Based upon this bioactivity, we hypothesized that curcumin presents molecular features that make it an excellent lead compound for the development of more effective inhibitors of Aβ aggregation. To explore this hypothesis, we screened a library of curcumin analogs and identified structural features that contribute to the anti-oligomerization activity of curcumin and its analogs. First, at least one enone group in the spacer between aryl rings is necessary for measureable anti-Aβ aggregation activity. Second, an unsaturated carbon spacer between aryl rings is essential for inhibitory activity, as none of the saturated carbon spacers showed any margin of improvement over that of native curcumin. Third, methoxyl and hydroxyl substitutions in the meta- and para-positions on the aryl rings appear necessary for some measure of improved inhibitory activity. The best lead inhibitors have either their meta- and para-substituted methoxyl and hydroxyl groups reversed from that of curcumin or methoxyl or hydroxyl groups placed in both positions. The simple substitution of the para-hydroxy group on curcumin with a methoxy substitution improved inhibitor function by 6-7-fold over that measured for curcumin.

## Introduction

It is estimated that approximately 20 million people worldwide currently suffer from age-related dementia caused by Alzheimer's Disease (AD). Individuals afflicted with AD suffer from a variety of unpredictable behaviors including loss in cognition, poor learning and memory, and severe mood changes. The prevalence of the pathology increases from 3% of the population at age 65 to 47% after the age of 85 [1]. The neuropathology of AD has been well studied over the past several decades. One of the earliest histological changes seen in the brains of AD patients is the deposition of amyloid-like plaques. The presence of amyloid plaques predisposes clinical symptoms of cognitive impairment suggesting that these abnormal brain deposits participate in events leading to the clinical presentation of dementia [2], [3], [4]. Formation of these plaques is thought to begin in the entorhinal complex and hippocampus, later progressing into the neocortex [5]. Disease progression is accompanied by a decrease in neural metabolic activity and an increase in neural cell death. These observations have led to the hypothesis that a reduction in amyloid plaque burden is expected to slow or halt the progression of AD and improve cognitive function.

Although many blood-borne proteins have been identified in amyloid plaques, the main constituent is a hydrophobic peptide called Aβ [6]. The Aβ peptide originates from what is believed to be normal processing of the amyloid precursor protein (APP). APP, a transmembrane protein, is cleaved in two successive proteolytic reactions to release Aβ peptide, which is either 40 or 42 amino acids in length depending on its intramembrane cleavage site. Once formed, it is thought that Aβ is cleared through normal drainage function of the cerebral spinal fluid (CSF) [7], [8], [9]. Aβ-related pathologies develop when free peptide, once reaching a critical concentration, forms insoluble oligomers which seed further aggregation eventually leading to the formation of characteristic amyloid lesions.

Current therapies for Alzheimer's disease focus largely on symptomatic aspects of the clinical pathology. Strategies include increasing cholinergic neurotransmission by administering acetylcholine esterase inhibitors (e.g. Tacrine or Donepezil) [10] and modulation of NMDA receptor activity by Memantine [11]. Although these therapies have shown a modest effect on slowing cognitive decline, they have yet to demonstrate any major impact on the progression of the disease. As an alternative to these therapies, prevention of Aβ aggregation has been attempted through use of small molecule inhibitors [12], [13]. From these efforts, a number of useful lead compounds have been identified such as sulfonated anions, benzofuran derivatives, as well as other polyphenol-based compounds [14], [15], [16], [17], [18]. However, the usefulness of these inhibitors has been limited due to their toxicity or their inability to cross the blood-brain barrier.

In contrast to these compounds, it was recently reported that the natural product curcumin, a non-toxic component of the spice turmeric, is capable of crossing the blood-brain barrier when injected into the circulation and reduce amyloid plaque burden *in vivo* in a transgenic mouse model [19], [20]. Curcumin is also capable of disaggregating preformed Aβ fibrils [21], [22]. Curcumin was less effective, however, when added to the diet [23], [24] indicating that its effectiveness *in vivo* has considerable room for improvement. Based upon its proven bioactive properties, it can be hypothesized that curcumin presents molecular features that make it an excellent lead compound for the development of more effective inhibitors of Aβ aggregation. Recently, investigators have begun to address this hypothesis by introducing modifications into the basic structure of curcumin and examining the effect of these changes on Aβ aggregation [25], [26], neuroinflammation [25] and Aβ-induced neurotoxicity [27]. Results from these investigations have shown that replacement of the 1,3-dicarbonyl moiety in curcumin with isosteric isoxazoles and pyrazoles generated compounds that inhibited g-secretase activity [28] and prevented both Aβ and Tau aggregation [26]. More modest changes in the curcumin structure still retained protective activity toward Aβ-induced neurotoxicity [27]; however, some changes, such as saturation of the 7-carbon linker to generate tetrahydrocurcumin, abolished Aβ aggregation inhibitory activity, but retained anti-neuroinflammation activity [25]. Although these findings clearly show that the base structure of curcumin can be modified without compromising certain properties of its bioactivity, none of the compounds tested show significant improvement as Aβ aggregation inhibitors when compared to native curcumin. To further explore if modifications to the native structure of curcumin can result in the identification of improved inhibitors of Aβ aggregation, we have generated chemical analogs of curcumin with various modifications and substitutions on the phenolic rings, varying degrees of unsaturation of the spacer between between aromatic rings, as well as compounds that contain either 5- or 7-carbon spacers to determine if spatial variations between phenols affects anti-Aβ aggregation activity [29]. We have identified several novel analogs of curcumin that are improved inhibitors of Aβ oligomerization.

## Materials and Methods

### Reagents and Materials

1,1,1,3,3,3-Hexafluoro-2-propanol (HFIP), dimethylsulfoxide (DMSO), fraction V bovine serum albumin and all buffer reagents were obtained from Sigma-Aldrich (St. Louis, MO). Tetramethylbenzidine (TMB) was purchased from Roche (Indianapolis, IN). Human Aβ(1–42) was purchased from AnaSpec (San Jose, CA). NUNC MaxiSorp ELISA plates were obtained from eBioscience (San Diego, CA). Monoclonal antibody 4G8 specific for human Aβ amino acids 17–24 and horseradish peroxidase (HRP)-conjugated 4G8 were purchased from Signet Labs (Dedham, MA). Synthesis of curcumin and analogs was previously reported [29].

### Preparation of monomeric Aβ(1–42) peptide

Aβ(1–42) peptide was dissolved in HFIP [30], [31] to a final concentration of 4 mg/ml and divided into 500 µg aliquots. Aliquots were dried under a stream of sterile N_2_ and stored at −20°C until use. Immediately preceding each experiment, aliquots were dissolved in DMSO to a final concentration of 1 mM. Solutions were sonicated for 15 min followed by heating at 60°C for an additional 15 min. Any unused peptide was discarded.

### Aß peptide oligomerization reactions

Aβ peptide from DMSO stock was diluted to the indicated concentrations either into phosphate buffered saline, pH 7.2 (PBS) alone or into test compound, pre-diluted into PBS. Stock solutions of all test compounds were made with DMSO for solvent compatibility. Dilutions were large enough to ensure that final DMSO concentrations were consistently <1% in the reaction mix. Reactions were incubated at 37°C for 24 h and then processed for capture ELISA.

### Capture ELISA for Aβ oligomer detection

NUNC Maxisorp high-binding ELISA plates were coated with mAb 4G8 diluted to 2 µg/ml in PBS for a minimum of 16 h at 4°C. After rinsing plates with PBS and blocking non-specific sites with PBS-T/B (PBS containing 0.1% Tween-20, 1% bovine serum albumin) for 1.5 h, Aβ peptide oligomerization reactions were added to wells and incubated with immobilized capture mAb for 2 h. Wells were rinsed three times with TBS-T (20 mM Tris-HCl, 150 mM NaCl, pH 7.4, 0.05% Tween-20) using a Biotek ELx50 automated plate washer. HRP-conjugated mAb 4G8 was added to wells at 1 µg/ml diluted into PBS-T/B and incubated at 23°C for 1 h. Unbound secondary antibody was removed by rinsing three times with TBS-T and bound antibody was measured following addition of TMB reagent. TMB reaction was terminated after ∼10 min with the addition of an equal volume of 1 M H_2_SO_4_. Absorbance was recorded at 450 nm with a reference wavelength of 650 nm using a Molecular Devices SpectraMax 384 Plus plate reader.

### Wst-1 assay

Microglial cells (100 µl containing 50,000 cells) were added to wells of a 96-well culture plate and incubated for 24 h at 37°C in a 5% CO_2_ incubator. Cells were then incubated with the indicated concentrations of curcumin or compound **2** and cells were incubated for an additional 24 h. Media was removed and Wst-1 reagent (diluted 1∶40 into complete phenol red-free media) was added to each well and the cultures were incubated at 37°C, 5% CO_2_ for 1 h. Absorbance was measured at 460 nm.

### Statistical analyses

All experimental protocols were carried out in at least triplicate points to determine mean values. Error bars represent standard deviation from mean values. Intra- and inter-assay variations for capture ELISAs were routinely ≤5%.

## Results

We have previously constructed a chemical library of curcumin-based analogs for the initial purpose of identifying the functional groups responsible for curcumin's anti-oxidant properties [29], [32]. This library includes compounds with the following variations on the curcumin structure. Some compounds have five carbon spacers between the aromatic rings instead of the seven carbon spacer of curcumin. The degree of unsaturation of the spacer is varied. The positions and number of the phenolic and methoxyl groups are varied and in some cases other groups are present. Some compounds also have substitutions at the central carbon of the spacer.

In order to perform large-scale screening of our analog library in a rapid, reproducible and cost-effective manner, we developed a novel ELISA-based assay to quantify oligomeric Aβ peptide [33]. Importantly, this assay clearly distinguishes the oligomeric conformation of the Aβ aggregate from the fibrillar form, which is important since the oligomeric form of Aβ aggregates is receiving increasing attention as a major factor responsible for synaptic dysfunction [34].

**Figure 1:**
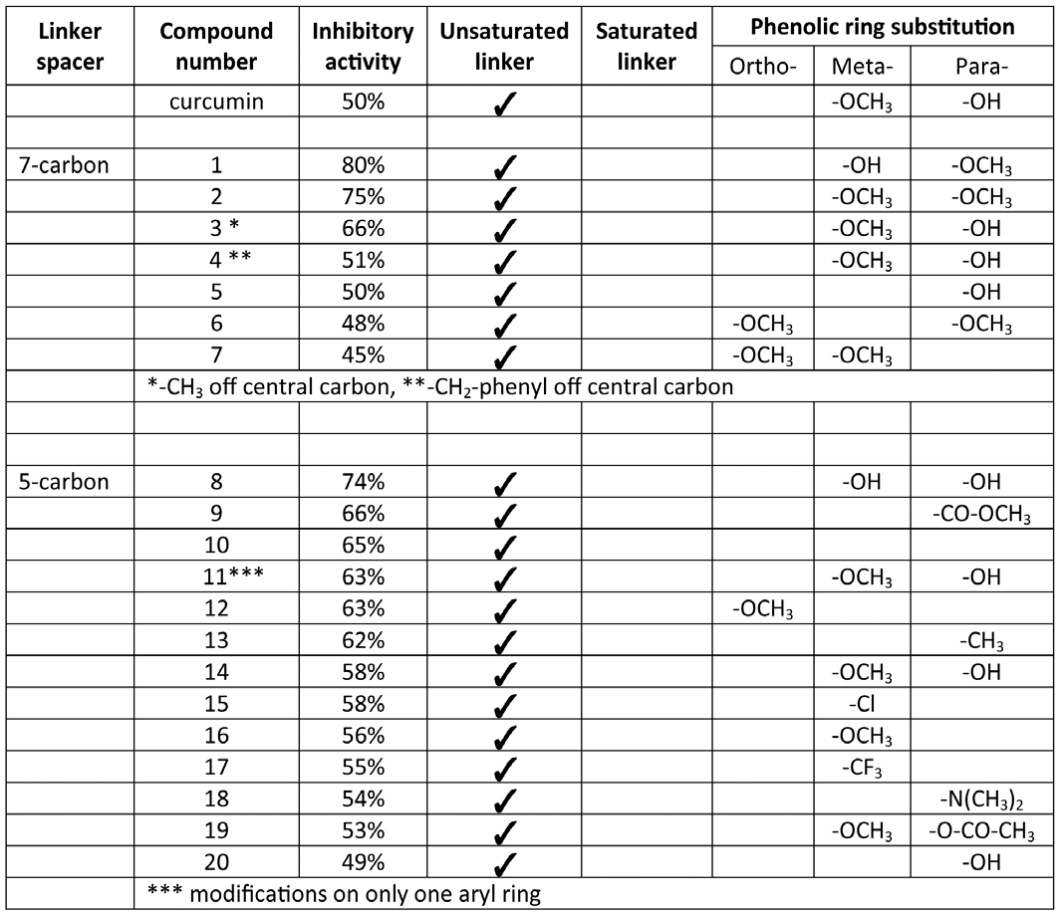
Structure/function assessment of curcumin analogs. Quantitative assessment of 20 curcumin analogs for inhibitory activity of Aβ aggregation and comparison with chemical substitutions made to the curcumin structure.

**Figure 2:**
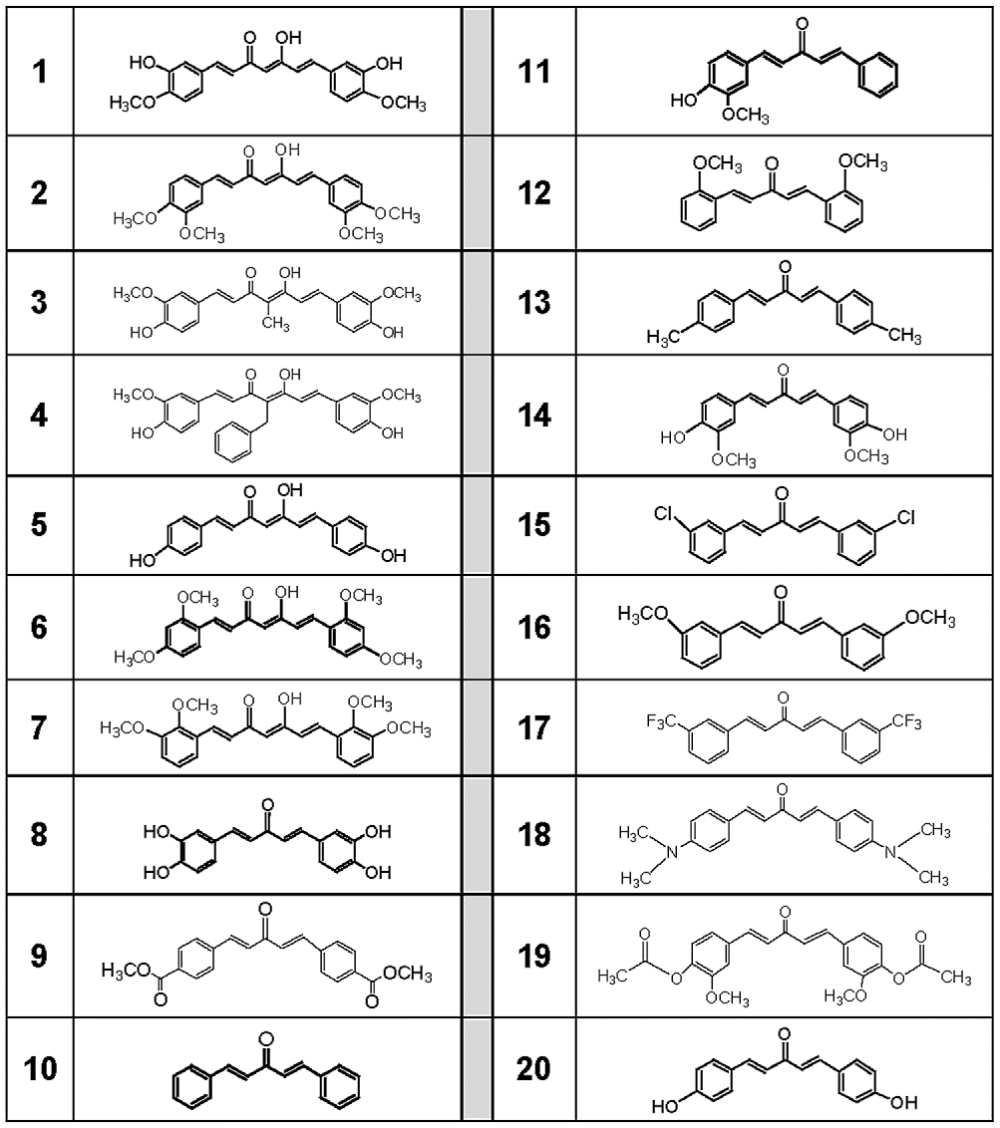
Structures of active analogs of curcumin. The analogs shown were identified as those that inhibited Aβ oligomerization equal to or better than curcumin (data obtained from Figure 1; inhibitory activity ≥50% within one S.D. of mean values). These analogs include 7 compounds from the 7-carbon series and 13 compounds from the 5-carbon series.

We first performed a general screen of our curcumin-based chemical analog library to identify if compounds are present in our library that are more effective than curcumin in preventing formation of Aβ oligomers. Analogs were tested for anti-Aβ aggregation activity at curcumin's IC_50_ value (1 µM) for Aβ oligomerization. This IC_50_ value for curcumin was previously established [33], [35]. For analog screening, monomeric Aβ peptide (200 nM) was incubated alone or with 1 µM curcumin or with 1 µM of the indicated analog. Following this incubation, oligomers were then quantified by capture ELISA. Analogs were identified that inhibited Aβ oligomerization equal to or better than curcumin **(** **Fig. 1**; Inhibitory activity ≥50% within one S.D. of mean values). A total of 20 compounds met this criteria and were used for a structure/function assessment (**Fig. 1**). Structures of the 20 analogs are shown in **Figure 2** and include 7 compounds from the 7-carbon series and 13 compounds from the 5-carbon series. Interestingly, all of these curcumin analogs have unsaturated linkers joining the phenolic rings, yet contain a variety of ring substitutions which likely dictates the quantitative differences measured in inhibitory function.

**Figure 3:**
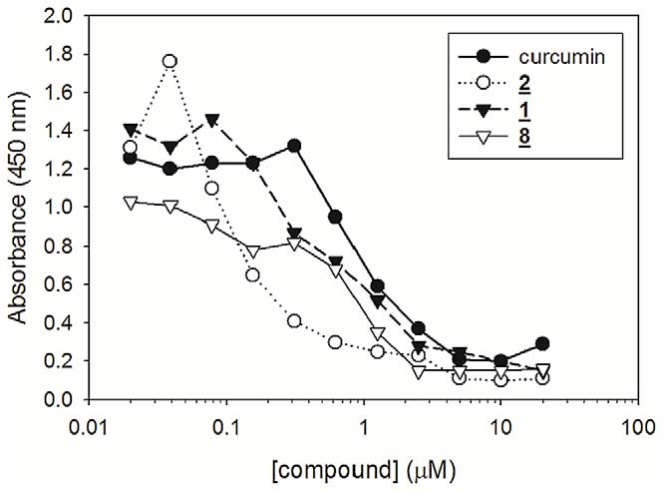
Quantitative comparison of curcumin with analogs 1, 2, and 8 as Aβ oligomerization inhibitors. Soluble Aβ monomeric peptide was prepared as described in methods and diluted to a final concentration of 200 nM directly into phosphate buffered saline (PBS), pH 7.4, or PBS containing the indicated concentrations of curcumin or analogs **1**, **2** or **8**. Reactions were incubated at 37°C for 24 h. Oligomers were quantified by capture ELISA. All reactions were prepared in triplicate to calculate mean values. Standard deviations from mean values were calculated and amounted to <5% for each experimental point.

We next selected our three best compounds (compounds **1,** **2,** and **8**) for dose-response studies. Compounds **1** and **8** both demonstrated anti-aggregation IC_50_ values slightly less than curcumin, 0.8 µM and 0.6 µM, respectively (**Fig. 3**). Compound **2** demonstrated markedly improved inhibitory capacity over that of curcumin with an IC_50_ value of 0.15 µM, making it the best lead compound identified in this analog library.

**Figure 4:**
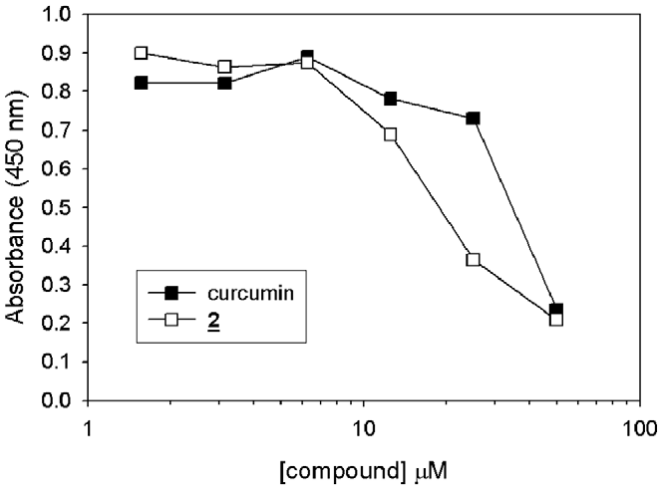
Quantitative assessment of cytotoxic effects of curcumin and compound 2 on murine microglial cells. Cultured microglial cells were incubate without or with the indicated concentrations of curcumin or compound 2 for 24 h. Cells were then incubated with Wst-1 reagent for 1 h, afterwhich absorbance was measured (460 nm). Values on graph represent mean values calculated from triplicate experimental points. Standard deviations from mean values were determined as <5% for each experimental point.

Since curcumin has been reported to demonstrate cytotoxicity in some cultured cell systems [36], we determined if our lead compound **2** showed equal, or perhaps reduced, levels of toxicity toward cells of neuronal origin. Murine microglial cells were incubated with varying concentrations of either curcumin or compound **2** for 24 hr and cell health was assessed by measuring cytoplasmic dehydrogenase activity. Consistent with previous results, curcumin demonstrated dose-dependent toxicity effects with an LD_50_ value of 40 uM (**Fig. 4**). Compound **2** also showed some toxicity toward microglial cells, however, its LD_50_ value is approximately 2-fold lower than that of curcumin. This finding shows that, in addition to improving on curcumin's anti-aggregation effects, compound **2** also shows reduced cytotoxicity as compared with curcumin. Most importantly, the IC_50_ value of compound **2** for anti-aggregation activity is well below its LD_50_ levels for cytotoxicity. This indicates that the dosage of compound **2** required for biologic activity is expected to be well below concentrations that might induce neuronal cytotoxicity; an important consideration to validate compound **2** as a viable lead compound.

## Discussion

Recent studies utilizing well established animal models have provided valuable insights on curcumin's role in AD [20], [37]. When administered as a dietary supplement, curcumin reduced Aβ deposition in aged APP(Swedish)-transgenic mice (Tg2576) demonstrating its ability to cross the blood-brain barrier in sufficient quantities to reduce amyloid burden. In vitro measurements have permitted a quantitative assessment of curcumin function by showing that it inhibits the formation of low-molecular weight Aβ oligomers and high-molecular weight fibrils with IC_50_ values of 1.0 µM and 0.8 µM, respectively [20]. These observations, among others, have helped to establish curcumin as one of the most promising lead compounds in recent years that offers real potential for reducing amyloid deposition in AD, and in doing so, halting or reversing disease progression. The goal of the present study was to identify and develop more effective aggregation inhibitors by capitalizing on the newly established inhibitory properties of curcumin. In order to achieve this goal, we have hypothesized that the base structure of native curcumin provides an excellent starting point to identify chemical analogs that have greater efficacy in reducing or preventing Aβ peptide oligomer formation, while improving upon the generally poor bioavailability of curcumin.

The low micromolar IC_50_ value for inhibition of Aβ oligomerization clearly shows curcumin's potent bioactivity both in vitro and in vivo, and yet, this value also indicates that there is much room for improvement. To identify improved inhibitors, we have examined our previously constructed chemical library of analogs [29] for inhibitors of Aβ oligomerization that are significantly improved over the bioactivity of curcumin. This library includes compounds with variations on carbon spacer length between phenolic rings (7- or 5-carbons in length), a variety of ring substitutions, as well as substitutions to the central methylene carbon of curcumin.

**Figure 5:**
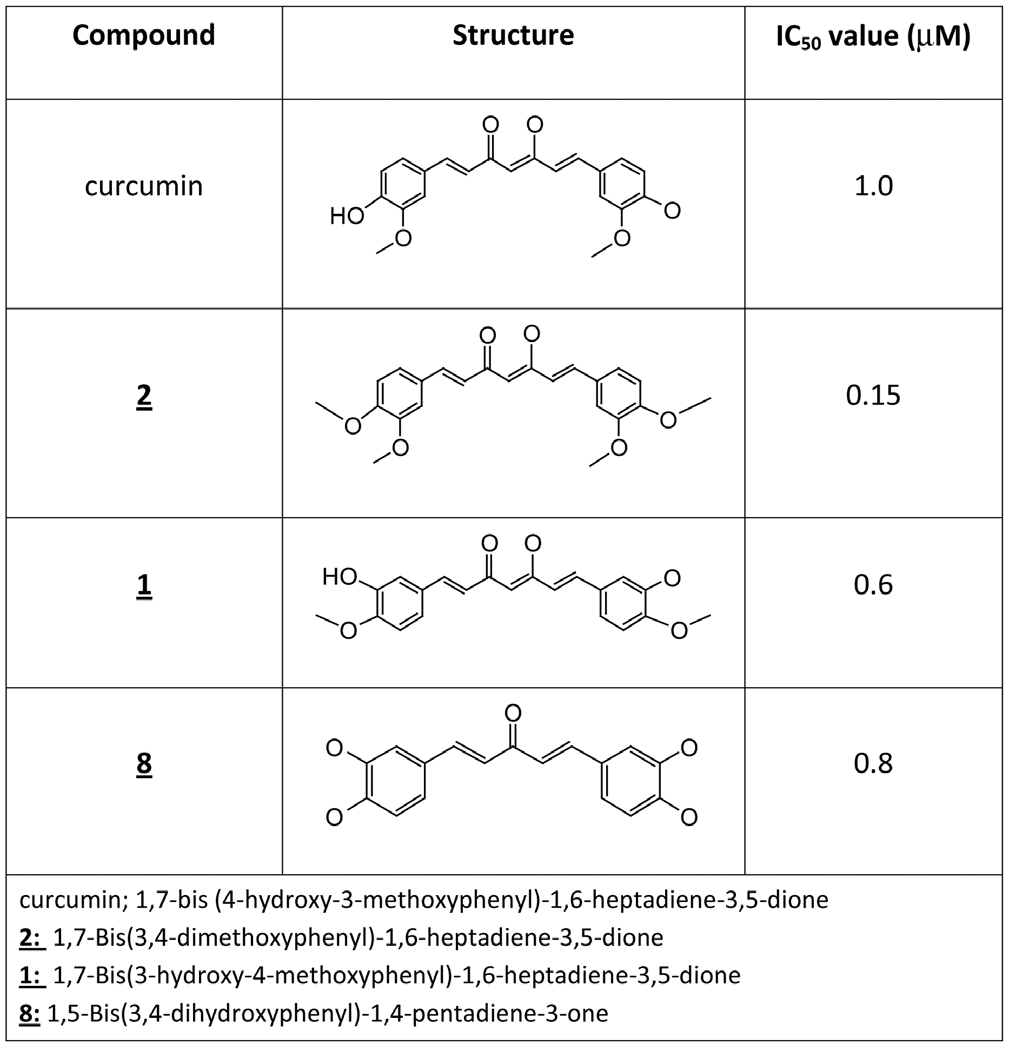
Comparison of chemical structures and IC_50_ values of analogs demonstrating improved bioactivity as Aβ oligomerization inhibitors. Compound **2** represents an improvement of approximately 6–7 fold over Aβ aggregation inhibitory activity of curcumin.

In general, our studies indicate that at least one enone group on the spacer is necessary for measureable anti- Aβ aggregation activity. The most striking feature among compounds in both the 7- and 5-carbon series listed in **Figure 1** is the presence of an α/β-unsaturated carbon spacer. None of the compounds with saturated spacers demonstrated inhibitory activity (data not shown), indicating that an unsaturated spacer between aryl rings is essential for anti- Aβ aggregation activity. A similar finding was reported by Begum, et al., when they compared the anti-amyloidogenic activities of dietary curcumin with that of tetrahydrocurcumin [25]. Further study of **Figure 1** reveals novel structure/function relationships with regard to specific substitutions to the aryl rings. Ortho-substitutions do not appear to contribute to improved inhibitor activity; however, maintaining methoxyl and hydroxyl substitutions in the meta- and para-positions on the aryl rings is necessary for comparable or improved inhibitory activity when measured against curcumin. In the 5-carbon series, one compound was significantly improved over that of curcumin, compound **8**, which has hydroxyl groups in both meta- and para-positions of the aryl rings (**Fig. 5**). The most improved inhibitors identified in the 7-carbon series have their meta- and para-substituted methoxyl and hydroxyl groups reversed from that of curcumin, as with compound **1**, or methoxyl groups placed in both positions, as with compound **2** (**Fig. 5**). The simple substitution of the para-hydroxy group on curcumin with a methoxy substitution (compound **2**) improved inhibitor function by 6-7-fold over that measured for curcumin, making compound **2** our most potent lead analog for anti-Aβ aggregation activity.

Additional challenges lie ahead to improve the bioactivity of our curcumin-derived analog in order to increase the therapeutic dose to the CNS. Questions in regard to bioavailability have plagued the use of curcumin as a potential therapeutic for a number of years [23]. Clinical trials have shown that the inherent bioavailability of orally administered curcumin is relatively low when factoring in intestinal absorption, liver metabolism and BBB penetrance [38]. However, in spite of these difficulties, dietary supplementation of curcumin administered to aged APP(Swedish)-transgenic mice (Tg2576) significantly lowered Aβ deposition in the CNS [20]. These findings clearly show that curcumin is able to enter the circulation and cross the BBB in sufficient quantities to reduce amyloid burden. To improve upon this property, we anticipate that the methoxy substitution on our lead compound **2** will decrease polarity and increase lipid membrane solubility thereby improving passive diffusion across the blood brain barrier (BBB) and access to the CNS [39], [40]. Similar observations have been made for other inhibitors of Aβ aggregation such as Chrysamine G [41]. In this study, the more lipophilic compound Chrysamine G was compared with Congo Red and found to readily cross the BBB in normal mice, achieving a brain∶blood ratio of greater than 10∶1. Moreover, metabolic inactivation poses other challenges to maintaining bioactivity. In this respect, the hydroxyl groups on curcumin are modified by enzymes found in the liver, kidney and intestinal mucosa [42] to form curcumin glucuronides and curcumin sulfates [43], [44]. The methoxy substitution for these hydroxyl groups on our lead compound **2** should prevent these glucuronide and sulfate additions and contribute to sustained bioactivity.

Proceeding from successful transgenic mouse studies [19], [22], [25], [45], human clinical trials have recently been initiated that are designed to examine the efficacy of dietary curcumin in slowing or reversing cognitive decline [46]. In general, curcumin studies have demonstrated that dietary administration of the compound in doses up to 12 g per day is well tolerated [46]; however, its effects on slowing or reversing cognitive decline have been modest at best and very often dependent on the stage of AD when treatment commences. For example, in an Asian study of 1,010 non-demented individuals, a small but statistically significant improvement in cognitive abilities was noted in a population that consumed curry more than once per month [47]. By contrast, in a more recent six-month randomized study, patients with moderate-to-severe Alzheimer's disease showed little or no measureable improvement when compared with placebo controls [48]. These clinical findings conflict with data obtained from curcumin-treated animal models and suggest challenges lie ahead in translating findings from rodent studies to human trials. Perhaps these challenges can be met by more clearly defining the objective of curcumin treatment; either as a preventative to delay or avert the onset of significant cognitive impairment in early stage AD patients or as a therapeutic aimed at reversing the clinical hallmarks of dementia found in more advanced stages. Thus far, the majority of rodent studies have been carried out by administering curcumin to animals prior to their developing AD pathologies, whereas the majority of human trials that have been attempted largely recruit individuals who are already symptomatic of AD and likely to have significant amyloid plaque burden. Reversing an already substantial plaque load may require multiple therapeutic modalities to supplement curcumin's bioactivity [49] or, alternatively, a more effective compound targeting Aβ plaque development such as the improved inhibitor presented here.
